# The effect of pharmaceutical companies’ marketing mix strategies on physicians prescribing practices in Jordan: a cross-sectional study

**DOI:** 10.1186/s12913-022-08664-1

**Published:** 2022-10-27

**Authors:** Dana Hisham Al Thabbah, Mohammad Salameh Almahairah, Abdallah Y Naser, Hamzeh Mohammad Alrawashdeh, Mosaab Araidah

**Affiliations:** 1 Management Information System Department, Faculty of Business, Isra University, Amman, Jordan; 2 Department of Applied Pharmaceutical Sciences and Clinical Pharmacy, Faculty of Pharmacy, Isra University, Amman, Jordan; 3 Department of Ophthalmology, Sharif Eye Centers, Irbid, Jordan; 4 Clinical Department, Triumpharmacy Centre, Amman, Jordan

**Keywords:** Jordan, Marketing mix, Physicians, Prescribing

## Abstract

**Background:**

Exploring the effect of different marketing mix strategies on physicians’ prescribing practices is important due to its positive effect on the management of patients’ diseases and improving the health status of individuals by promoting the use of the most cost-effective and safe treatment for patients.

**Aim:**

This study aimed to assess the perceived influence of the four pharmaceutical marketing mix strategies (product, price, place, and promotion) on physicians’ prescribing practices in Jordan.

**Method:**

A quantitative survey study was conducted from May to November 2021 on practising physicians in Jordan. This research utilised a previously validated questionnaire developed by Hailu et al. The convenience sampling technique was used to recruit the study participants. The population of the study was practising physicians from the public and private sectors in Jordan. Any physician who was licensed to practice medicine in Jordan and actively engaged in patient care was considered eligible. The minimum sample size required was 379 participants, which was calculated based on a population size of 35,000 physicians in Jordan. Student t-test/One-way independent-measures ANOVA was used to compare the mean scores (indicating being affected by marketing mix elements between different demographic groups) after performing log transformation to restore the normality of the data. For the binary regression analyses, the dependent variable was the median score for each of the marketing mix elements. For each sub-scale and the overall scale, the median score was used to define the dummy variable used in the binary regression analysis. The study protocol was approved by the Scientific Research Ethics Committee at Isra University (SREC/21/06/005).

**Results:**

A total of 315 physicians participated in the study. Overall, participating physicians showed moderate to high influence from marketing mix elements, with a median score of 141.0 (IQR: 118.0-156.0) out of 185, representing 76.2%. The lowest median score was found for the promotional tools used by pharmaceutical companies, with a median score of 48.5 (IQR: 40.0–56.0) out of 70, representing 69.3%. The highest median score found was for the pricing strategy implemented by pharmaceutical companies, with a median score of 25.0 (IQR: 18.8–28.0) out of 30, representing 83.3%. Working in private sector settings was an important predictor that increased the probability of physicians’ prescribing practices being influenced by marketing mix elements (OR: 1.57; (95%CI: 1.00-2.47)), (p ≤ 0.05).

**Conclusion:**

Physicians in our study were highly affected by marketing mix strategies, specifically price strategy. Policymakers should guarantee a balanced relationship with pharmaceutical companies and physicians. We should make sure that promotion strategies have a positive impact on patients’ health. The government is advised to decrease the taxes on medications to decrease the overall cost for patients.

## Background

The pharmaceutical sector has a significant impact on world healthcare. Competition is beneficial because it forces businesses to deliver higher-quality goods and services at lower costs. Competition in the pharmaceutical sector can stimulate brand companies to develop new and improved treatments while encouraging generic companies to offer less expensive alternatives [1]. The pharmaceutical industry is regarded as one of the world’s most profitable and inventive industries [2]. It is a stable business that is not vulnerable to economic crises, yet it must meet the requirements of humanity in terms of global health concerns and social aspirations.

Products from local pharmaceutical firms are confronted with multiple issues, the most serious of which is competition. Foreign pharmaceutical companies are increasingly accepted in Jordan’s medicine and pharmaceutical market. Many major foreign pharmaceutical companies have established strong brands and are focusing on developing ties with doctors. Marketing is one of the backbones of pharmaceutical companies.

Medicines can be used to treat acute illnesses and autoimmune reactions, relieve symptoms, and prevent future illness. Any decision to use a medicine, however, requires assessing potential advantages against potential risks. A person requires knowledge of the purpose of the therapy, how it works, how to use it appropriately, the possibility of benefits and risks, how the drug compares to other available treatment alternatives or the decision not to treat, and relative cost-effectiveness to make an informed decision. The quality of the information provided with drugs might be the difference between “a poison and a cure” or between beneficial use and one that is most likely to cause harm. Incorrect data on diseases and disease risks, which is just as important as information on medicines, can cause harm if patients seek medical care when it is not essential, resulting in wasteful medicine use and the risk of drug-induced injury [3].

Pharmaceutical demand is influenced by many factors, such as health awareness and the quality of treatment provided [4]. The pharmaceutical industry around the world is heavily involved in active drug promotion activities to change doctors’ prescription practices and encourage patients to self-administer drugs. Competitiveness is one of the most important survival requirements for service and product suppliers [5].

On average, pharmaceutical companies spend 20% or more of their income on marketing [6]. An estimated 84% of pharmaceutical marketing efforts are directed at doctors. Medical promotion, salespersons as medical representatives, and detail personnel are the most important participants in marketing medicines to physicians [7]. Pharmaceutical detailing is a strategy used by pharmaceutical companies to inform physicians about a vendor’s products in an effort to change the physician’s prescribing practices and increase the market share for a particular drug [8]. Through sales calls, pharmaceutical sales personnel are trained to educate physicians and other healthcare professionals. Pharmaceutical detailing has a wide range of benefits, including raising sales, enhancing patient outcomes, promoting clinical research, and enhancing physician knowledge [8].

Pharmaceutical promotion enhances the health of people, families, communities, states, and the nation by encouraging healthcare providers to use the best treatment options available. On the other hand, it may have a negative influence as it might increase the probability of overtreatment, promoting improper substitutes and medication misuse [9].

Although many researchers have looked into various hypotheses of marketing’s influence on physicians, a need remains to be filled [6]. Research on the relationship between pharmaceutical companies and physicians is a comparatively new and sensitive topic drawing momentous attention in the Middle East. Pharmaceutical companies’ spending continues to escalate, and drug safety issues have become more common. Physician-directed outreach efforts have come under mounting public scrutiny [10]. As a result, pharmaceutical companies must develop strategic marketing methods that do not compromise the ethical code of conduct. They must comprehend how their marketing strategies impact physicians’ prescribing practices [11, 12]. To the best of our knowledge, no studies in Jordan or the Middle East region have explored the effect of all marketing mix strategies on physicians’ prescribing practices. Previous literature shows that few studies have investigated the impact of the four pharmaceutical marketing strategies (product, price, place, and promotion) on physicians’ prescribing practices in developing countries, specifically in the Middle East region. Previous studies focused on specific marketing mix strategies such as promotion tools [9, 11–14]. Changing prescribing practices could ultimately have a positive or negative impact on patients’ health. By promoting the best treatment options for healthcare professionals, pharmaceutical companies’ marketing strategies could improve the health status of individuals and the whole community. At the same time, they could have a negative impact by promoting potential overtreatment, cost-ineffective substitutions, and potential misuse. Therefore, the aim of this study was to determine the factors that influence physicians’ prescribing practices according to the four pharmaceutical marketing mix strategies (product, price, place, and promotion) in Jordan.

## Methods

### Study design

A quantitative survey study was conducted from May to November 2021 on practising physicians in Jordan.

### Study participants

The population of the study is practising physicians from the public and private sectors in Jordan. The study’s participants were physicians from any specialty, including specialists and general practitioners. Any physician who is licensed to practice medicine in Jordan and actively engaged in patient care was considered eligible. These inclusion criteria were clearly mentioned in the invitation letter for the survey.

### Participants’ recruitment

The convenience sampling technique was utilised in this research. Practicing physicians who were easily accessible and willing to participate in this research were approached and invited to participate in the study. The questionnaire was administered to the physicians who agreed to participate after explaining the study’s objectives. A participants’ information sheet was provided for further clarification of the study. In addition, they were informed that completing the questionnaire was considered to be written consent and an agreement to take part in the study. This involved the distribution of online and paper-based surveys to the physicians (295 online surveys (93.7%) and 20 paper-based surveys (6.3%)). All Jordanian physicians who are currently registered were sent the link to the electronic online questionnaire at their provided email addresses, which were received from the Jordanian Medical Association.

### Data collection

This study utilised a previously validated questionnaire developed by Hailu et al. (2021) [12], which explored factors that influence physicians’ prescribing practices by pharmaceutical marketing mix strategies. The socio-demographic characteristics of physicians that affect their influence by pharmaceutical marketing mix strategies were also investigated. The questionnaire included demographic questions and 5-point Likert scale questions. The Likert-question answers ranged from “strongly disagree” to “strongly agree”. The questionnaire was comprised mainly of four sections. The first section investigated the impact of promotional tools (questions 1–14), in which participants were asked whether promotional tools motivate them to prescribe a specific product for a drug company. In this section, the participants were asked about participating in company-sponsored continual medical education, information from medical representative, frequent visits of medical representative, sales calls made by pharmaceutical companies, free drug samples given by pharmaceutical company, information from promotional drug brochures, different gifts from pharmaceutical company, participating pharmaceutical company-sponsored entertainments/recreational events, sponsorship for travel in conference, subscription of journals with direct mail, invitation to visit a pharmaceutical manufacturing plant, personal relationship to company, product launch meeting, and public relation of pharmaceutical company. The second section explored the influence of product strategy (questions 15–24) and asked the participants whether product strategy motivate them to prescribe a certain product for a drug company. In this section, the participants were asked about country of pharmaceutical product manufacturer, image of pharmaceutical company, supportive evidence of the efficacy of the medicine given by pharmaceutical company, release of new innovations or combinations of drugs, form of delivery of the medicine, ease to remember brand names, reputation of the source of medicine, quality of medicine, fixed-dose packaging of the product, and full therapy packaging. The third section explored the influence of distribution strategies (questions (25–31) and asked the participants whether distribution strategy motivate them to prescribe a certain product for a drug company. In this section, the participants were asked about pharmaceutical product availability, inclusion of medicine in the hospital medicine list, availability of local agent (importer/distributor) representing the principal company, availability of real-time product information from distribution intermediaries, presence of sole supplier, fast deliveries with special storage and distribution of medicines, and reverse pharmaceutical (product recall). The fourth section explored the influence of pricing strategies (questions 32–37) and asked the participants whether pricing strategy motivate them to prescribe a certain product for a drug company. In this section, the participants were asked about disclosure of actual price of the product, price discounts technique for the product, price of the drug and effectiveness of therapy, price of medication in relation to quality, price competition among pharmaceutical company, and price for full course therapy.

### Sample size

The target sample size was estimated using a population size of 35,000 physicians in Jordan [15]. With a confidence interval of 95%, a standard deviation of 0.5, and a margin of error of 5%, the minimum required sample size was 379 participants.

$N = \,\frac{{{Z^2} * {p^ \wedge }\left( {1 - {p^ \wedge }} \right)}}{{{\varepsilon ^2}}}$ ; z is the z score; ε is the margin of error; N is the population size; p̂ is the population proportion

### Statistical analysis

Continuous variables were presented as median (interquartile range) as the data was not normally distributed, which was confirmed through histogram and skewness measures (-1.45). Categorical variables were presented as frequency and percentage. Binary logistic regression was conducted to identify demographic characteristics that influence physicians’ prescribing practices through different marketing mix strategies. The median score for each subscale and the overall scale was used to identify the dummy variables used in the binary regression analyses. Student t-test/One-way independent-measures ANOVA was used to compare the mean scores (score reflecting a marketing mix element(s) influence ) between different demographic groups after performing log transformation to restore the normality of the data. For the binary regression analyses, the dependent variable was the median score for each of the marketing mix elements (each subscale and the overall scale). For each sub-scale (price, place, promotion, and product) and the overall scale (total score for all marketing mix elements), the median score (for each subscale and the overall scale) was used to define the dummy variable used in the binary regression analysis. Any participants who scored above the median score was given the weight of 1 (yes) to define him/her as being influenced by marketing mix element(s) in terms of prescribing practices, and otherwise was given the weight of zero (no). Consequently, the highest attainable score would be 185. Cronbach’s alpha test was used to explore the reliability of the questionnaire. Statistical Package for Social Science Software version 27 was used to perform all statistical analyses. A confidence interval of 95% (p ≤ 0.05) was applied to represent the statistical significance of the results, and 5% was assigned as the level of significance.

### Reliability of the study tool

Table 1 shows the internal consistency of the questionnaire presented through Cronbach’s alpha measures. The Cronbach’s alpha measures ranged between 0.823 and 0.899 for the four sub-scales (product, price, place, and promotion), which reflects good internal consistency. The overall Cronbach’s alpha measure for the whole questionnaire was 0.930. This demonstrates excellent internal consistency for the questionnaire tool among our study sample.

**Table 1:** Cronbach’s alpha measure for the questionnaire tool

Marketing mix element	Number of items per sub-scale	Cronbach’s alpha measure
Promotional tools	14	0.899
Product strategy	10	0.868
Distribution strategy	7	0.823
Pricing strategy	6	0.823
**Overall scale**	37	0.930

A pilot study was conducted on 10 physicians who have met the inclusion criteria to confirm their understanding of the questionnaire and whether it is measuring what we are aiming to measure. The physicians confirmed the content and face validity of the questionnaire and found it clear and easy to measure the effect of marketing mix elements on their prescribing practices.

## Results

### Demographic characteristics of the participating physicians

A total of 315 physicians participated in the study. Of these, 63.2% were male. The highest frequency of the age group was 23–30 years (34.6%). Physicians who are specialists were the highest proportion (65.1%). Physicians with less than five years of experience were the most frequent (41.3%). The highest frequency of country of first degree in medical education was Jordan (63.2%), and 40.3% had Jordan as their country of specialisation. Over a third of respondents were general practitioners (34.9%), followed by internists (14.3%), paediatricians and ophthalmologists (9.2%). Nearly half (44.2%) were working in the public sector. Table 2 describes the demographic characteristics of the participating physicians.

**Table 2:** Demographic characteristics of the participating physicians

Demographic variable	Frequency (%)
**Gender**	
Male	199 (63.2%)
**Age category**	
23–30 years	109 (34.6%)
31–35 years	84 (26.7%)
36–40 years	35 (11.1%)
41–45 years	28 (8.9%)
46–50 years	14 (4.4%)
51 years and above	45 (14.3%)
**Year of experience**	
Less than 5 years	130 (41.3%)
6–10 years	88 (27.9%)
More than 10 years	97 (30.8%)
**Medical education**	
General practitioner	110 (34.9%)
Specialised physician	205 (65.1%)
**Country of first degree in medical education**	
Jordan	199 (63.2%)
Outside Jordan	116 (36.8%)
**Speciality**	
Not specialised (general practitioner)	110 (34.9%)
Internist	29 (14.3%)
Surgeon	8 (3.8%)
Gynaecologist	16 (7.9%)
Paediatrician	19 (9.2%)
Dermatologist	2 (1.0%)
Orthopaedics	8 (3.8%)
Resident	17 (8.3%)
ENTs	3 (1.6%)
Ophthalmologist	19 (9.2%)
Other	84 (41.0%)
**Country of speciality in medical education**	
Not specialised (general practitioner)	110 (34.9%)
Jordan	127 (40.3%)
Outside Jordan	78 (24.8%)
**Practice settings**	
Public healthcare sector	139 (44.1%)
Private healthcare sector	130 (41.3%)
Both settings	46 (14.6%)

### The impact of marketing mix elements on physicians’ prescribing practices

Overall, participating physicians showed moderate to high influence by marketing mix elements, with a median score of 141.0 (IQR: 118.0-156.0) out of 185, representing 76.2% of the maximum obtainable score for this sub-scale. The lowest median score was found for the promotional tools used by pharmaceutical companies, with a median score of 48.5 (IQR: 40.0–56.0) out of 70, representing 69.3% of the maximum obtainable score for this sub-scale. The median scores for product strategy, distribution strategy, and pricing strategy were comparable, ranging between 80.0% and 83.3% of the maximum obtainable score for each sub-scale. The highest median score found was for the pricing strategy implemented by pharmaceutical companies, with a median score of 25.0 (IQR: 18.8–28.0) out of 30, representing 83.3% of the maximum obtainable score for this sub-scale. See Table 3.

**Table 3:** Median score for the influence of each marketing mix element on physicians’ prescribing practices

Marketing mix element	Number of items per sub-scale	Maximum obtainable score for the sub-scale	Median score (IQR)	Median score out of the maximum obtainable score (%)
Promotional tools	14	70	48.5 (40.0–56.0)	69.3%
Product strategy	10	50	41.0 (35.0–45.0)	82.0%
Distribution strategy	7	35	28.0 (22.0–32.0)	80.0%
Pricing strategy	6	30	25.0 (18.8–28.0)	83.3%
**Overall scale**	37	185	141.0 (118.0-156.0)	76.2%

### The impact of promotion (promotional tools) on physicians’ prescribing practices

The most commonly agreed-upon promotional tools in terms of affecting physicians’ prescribing practices were participating in company-sponsored continual medical education (81.0%), product launch meetings (76.0%), and sponsorship for travel to conferences (73.4%), respectively. Table 4 shows the distribution of the degree to which physicians reported being affected by each promotional tool. The lowest agreement was for personal relationship to company (36.2%).

**Table 4:** Distribution of the degree to which physicians are being affected by each promotional tool

No	Description	Strongly agree	Agree	Neutral	Disagree	Strongly disagree
1	Participating in company-sponsored continual medical education	32.5%	48.5%	13.9%	3.6%	1.5%
2	Information from medical representative	19.7%	31.0%	21.9%	5.8%	21.5%
3	Frequently visits of medical representative	16.1%	36.1%	24.8%	16.4%	6.6%
4	Sales calls made by pharmaceutical companies	12.0%	46.7%	21.5%	14.2%	5.5%
5	Free drug samples given by pharmaceutical company	27.0%	44.2%	17.2%	6.9%	4.7%
6	Information from promotional drug brochures	25.9%	39.8%	22.6%	9.9%	1.8%
7	Different gifts from pharmaceutical company	12.4%	25.9%	26.3%	15.7%	19.7%
8	Participating pharmaceutical company-sponsored entertainments/recreational events	19.3%	30.7%	21.9%	15.7%	12.4%
9	Sponsorship for travel in conference	39.1%	34.3%	13.5%	5.1%	8.0%
10	Subscription of journals with direct mail	36.1%	35.8%	17.2%	6.6%	4.4%
11	Invitation to visit a pharmaceutical manufacturing plant	25.5%	38.0%	19.0%	11.3%	6.2%
12	Personal relationship to company	12.8%	23.4%	27.7%	19.7%	16.4%
13	Product launch meeting	29.6%	46.4%	16.1%	4.0%	4.0%
14	Public relation of pharmaceutical company	13.5%	40.9%	32.1%	9.1%	4.4%

### The impact of the product (product strategy) on physicians’ prescribing practices

The most commonly agreed-upon product strategies in terms of affecting physicians’ prescribing practices were quality of medicine (91.7%), supportive evidence of the efficacy of the medicine given by the pharmaceutical company (88.6%), and the release of innovations or combinations of drugs (86.8%), respectively. Table 5 shows the distribution of the degree to which physicians reported being affected by each product strategy. The lowest agreement was for the country of pharmaceutical product manufacturer (72.9%).

**Table 5:** Distribution of the degree to which physicians are being affected by each product strategy

No	Description	Strongly agree	Agree	Neutral	Disagree	Strongly disagree
1	Country of pharmaceutical product manufacturer	24.2%	48.7%	20.4%	4.2%	2.6%
2	Image of pharmaceutical company	24.2%	50.2%	20.0%	3.4%	2.3%
3	Supportive evidence of the efficacy of the medicine given by pharmaceutical company	50.9%	37.7%	8.3%	2.3%	0.8%
4	Release of new innovations or combinations of drugs	45.7%	41.1%	10.2%	2.6%	0.4%
5	Form of delivery of the medicine	38.1%	41.5%	15.8%	3.0%	1.5%
6	Easy to remember brand names	40.0%	34.7%	18.9%	5.3%	1.1%
7	Reputation of the source of medicine	44.2%	40.8%	12.5%	2.3%	0.4%
8	Quality of medicine	72.1%	19.6%	6.0%	1.5%	0.8%
9	Fixed-dose packaging of the product	34.7%	42.6%	17.0%	2.6%	3.0%
10	Full therapy packaging	44.2%	39.2%	13.2%	0.8%	2.6%

### The impact of place (distribution strategy) on physicians’ prescribing practices

The most commonly agreed-upon distribution strategies in terms of affecting physicians’ prescribing practices were the inclusion of the medicine in the hospital medicine list (89.8%), pharmaceutical product availability (89.4%), and the availability of real-time product information from distribution intermediaries (84.7%). Table 6 shows the distribution of the degree to which physicians reported being affected by each distribution strategy. The lowest agreement was for the presence of a sole supplier (38.4%).

**Table 6:** Distribution of the degree to which physicians are being affected by each distribution strategy

No	Description	Strongly agree	Agree	Neutral	Disagree	Strongly disagree
1	Pharmaceutical product availability	53.7%	35.7%	9.0%	0.8%	0.8%
2	Inclusion of medicine in the hospital medicine list	51.0%	38.8%	7.8%	1.2%	1.2%
3	Availability of local agent (importer/distributor) representing the principal company	43.9%	36.9%	15.3%	2.7%	1.2%
4	Availability of real-time product information from distribution intermediaries	40.4%	44.3%	11.8%	2.0%	1.6%
5	Presence of sole supplier	13.7%	24.7%	38.4%	16.1%	7.1%
6	Fast deliveries with special storage and distribution of medicines	50.2%	33.7%	12.2%	2.7%	1.2%
7	Reverse pharmaceutical (product recall)	45.9%	38.4%	10.6%	2.7%	2.4%

### The impact of price (pricing strategy) on physicians’ prescribing practices

The most commonly agreed-upon pricing strategies in terms of affecting physicians’ prescribing practices were the price of the drug and effectiveness of therapy (90.0%), the price for full course therapy (87.6%), and the price of medication concerning quality (87.2%). Table 7 shows the distribution of the degree to which physicians reported being affected by each pricing strategy. The lowest agreement was for price competition among pharmaceutical companies (78.4%).

**Table 7:** Distribution of the degree to which physicians are being affected by each pricing strategy

No	Description	Strongly agree	Agree	Neutral	Disagree	Strongly disagree
1	Disclosure of actual price of the product	54.0%	30.4%	10.8%	3.2%	1.6%
2	Price discounts technique for the product	38.4%	42.8%	15.2%	1.6%	2.0%
3	Price of the drug and effectiveness of therapy	61.6%	28.4%	7.2%	0.8%	2.0%
4	Price of medication in relation to quality	57.6%	29.6%	8.8%	1.6%	2.4%
5	Price competition among pharmaceutical company	46.0%	32.4%	16.8%	3.6%	1.2%
6	Price for full course therapy	58.4%	29.2%	8.0%	3.2%	1.2%

### The association between physicians’ demographics and being influenced by marketing mix elements in terms of prescribing practices

A binary logistic regression analysis was conducted to identify demographic factors affecting the influence of different marketing mix strategies on physicians’ prescribing practices. See Table 8. Working in private sector settings was an important predictor that increased the probability of physicians’ prescribing practices being influenced by marketing mix elements (OR: 1.57; (95%CI: 1.00-2.47)), (p ≤ 0.05).

**Table 8:** Factors affecting physicians influence by different marketing mix strategies

Demographic variable	Odds ratio of being influenced by all marketing mix elements (95%CI)	Odds ratio of being influenced by promotional tools (95%CI)	Odds ratio of being influenced by product strategy (95%CI)	Odds ratio of being influenced by distribution strategy (95%CI)	Odds ratio of being influenced by pricing strategy (95%CI)
**Gender**					
Male (Reference category)	1.00	1.00	1.00	1.00	1.00
Female	0.79 (0.50–1.25)	0.81 (0.51–1.28)	0.66 (0.42–1.04)	0.87 (0.55–1.38)	0.71 (0.45–1.12)
**Age category**					
23–30 years (Reference category)	1.00	1.00	1.00	1.00	1.00
31–35 years	0.88 (0.54–1.45)	1.23 (0.74–2.02)	0.99 (0.60–1.63)	1.26 (0.76–2.08)	1.31 (0.79–2.18)
36–40 years	1.31 (0.65–2.67)	1.07 (0.53–2.17)	1.48 (0.72–3.03)	1.38 (0.67–2.82)	0.67 (0.33–1.36)
41–45 years	1.81 (0.81–4.06)	1.92 (0.86–4.30)	0.94 (0.43–2.04)	1.20 (0.55–2.63)	0.96 (0.44–2.08)
46–50 years	0.96 (0.33–2.79)	1.01 (0.35–2.94)	0.94 (0.32–2.75)	1.19 (0.40–3.52)	1.11 (0.38–3.29)
51 years and above	1.23 (0.65–2.32)	1.06 (0.56-2.00)	1.87 (0.97–3.60)	1.39 (0.73–2.65)	1.16 (0.61–2.20)
**Year of experience**					
Less than 5 years (Reference category)	1.00	1.00	1.00	1.00	1.00
6–10 years	1.14 (0.69–1.86)	1.48 (0.90–2.42)	0.92 (0.57–1.51)	1.09 (0.67–1.79)	1.13 (0.69–1.86)
More than 10 years	1.23 (0.76–1.98)	1.10 (0.68–1.78)	1.84 (1.13–3.01)*	1.40 (0.86–2.27)	1.06 (0.66–1.72)
**Medical education**					
General practitioner (Reference category)	1.00	1.00	1.00	1.00	1.00
Specialized physician	0.67 (0.21–2.17)	0.89 (0.56–1.41)	1.29 (0.81–2.05)	1.08 (0.68–1.71)	1.26 (0.79-2.00)
**Country of first degree in medical education**					
Jordan (Reference category)	1.00	1.00	1.00	1.00	1.00
Outside Jordan	1.01 (0.95–1.08)	1.33 (0.84–2.10)	1.29 (0.81–2.05)	1.15 (0.72–1.82)	0.93 (0.59–1.47)
**Specialty**					
Not specialized (general practitioner) (Reference category)	1.00	1.00	1.00	1.00	1.00
Internist	0.73 (0.39–1.38)	0.70 (0.37–1.32)	0.80 (0.43–1.51)	0.44 (0.23–0.84)*	0.56 (0.30–1.06)
Surgeon	1.10 (0.69–1.74)	1.43 (0.44–4.60)	2.94 (0.78–11.08)	0.88 (0.28–2.80)	0.58 (0.18–1.87)
Gynecologist	2.16 (0.90–5.15)	1.56 (0.68–3.60)	0.86 (0.38–1.95)	1.98 (0.83–4.74)	1.85 (0.77–4.42)
Pediatrician	1.20 (0.56–2.58)	0.69 (0.32–1.49)	1.18 (0.55–2.54)	1.28 (0.59–2.79)	1.03 (0.48–2.21)
Dermatologist	0.48 (0.04–5.29)	2.03 (0.18–22.57)	1.90 (0.17–21.17)	1.78 (0.16–19.85)	0.41 (0.04–4.59)
Orthopedics	2.98 (0.79–11.22)	1.43 (0.44–4.59)	2.94 (0.78–11.08)	2.75 (0.73–10.37)	4.35 (0.94–20.20)
Resident	0.57 (0.25–1.30)	1.01 (0.45–2.25)	0.39 (0.16–0.93)*	0.63 (0.28–1.41)	0.82 (0.37–1.83)
ENTs	0.63 (0.10–3.84)	1.52 (0.25–9.22)	1.43 (0.24–8.64)	1.34 (0.22–8.10)	1.25 (0.21–7.60)
Ophthalmologist	0.61 (0.34–1.10)	1.01 (0.57–1.80)	0.66 (0.37–1.18)	0.86 (0.48–1.54)	0.95 (0.53–1.70)
Others	1.13 (0.67–1.91)	0.84 (0.50–1.43)	0.90 (0.53–1.51)	1.10 (0.65–1.86)	0.82 (0.48–1.38)
**Country of specialty in medical education**					
Outside Jordan (Reference category)	1.00	1.00	1.00	1.00	1.00
Jordan	0.58 (0.31–1.08)	0.40 (0.21–0.77)**	1.40 (0.89–2.21)	1.18 (0.75–1.87)	1.24 (0.79–1.96)
**Practice settings**					
Public healthcare sector (Reference category)	1.00	1.00	1.00	1.00	1.00
Private healthcare sector	1.57 (1.00-2.47)*	1.01 (0.65–1.58)	2.12 (1.34–3.36)**	1.90 (1.20–3.01)**	1.38 (0.88–2.17)
Both settings	1.05 (0.56–1.97)	1.37 (0.73–2.57)	1.15 (0.61–2.15)	0.78 (0.42–1.47)	1.88 (0.97–3.64)

*p ≤ 0.05; **p ≤ 0.01

### The association between physicians’ demographics and being influenced by promotional tools

In terms of predictors of being influenced by promotional tools provided by pharmaceutical companies, the logistic regression analysis identified that physicians who received their specialised education locally in Jordan were less likely to be affected by promotional tools provided by pharmaceutical companies (OR: 0.40; 95%CI: 0.21–0.77) (p ≤ 0.01).

### The association between physicians’ demographics and being influenced by product strategies

In terms of predictors of being influenced by product strategies applied by pharmaceutical companies, the logistic regression analysis identified physicians who have more than ten years of experience and those who work in the private sector as more likely to be affected by product strategies compared to others, with (OR: 1.84; (95%CI: 1.13–3.01)) and (OR: 2.12; (95%CI: 1.34–3.36)) (p ≤ 0.05), respectively. On the other hand, resident physicians were less likely to be influenced (OR: 0.39; (95%CI: 0.16–0.93)), (p ≤ 0.05).

### The association between physicians’ demographics and being influenced by distribution strategies

In terms of predictors of being influenced by distribution strategies (place) applied by pharmaceutical companies, the logistic regression analysis identified that physicians who work in the private sector are more likely to be influenced by pharmaceutical companies’ distribution strategies (OR: 1.90; (95%CI: 1.20–3.01)) (p ≤ 0.01). However, internists were less likely to be influenced (OR: 0.44; (95%CI: 0.23–0.84)) (p ≤ 0.05).

### The association between physicians’ demographics and being influenced by pricing strategies

In terms of predictors of being influenced by pricing strategies applied by pharmaceutical companies, the difference between physicians from different demographic groups in terms of the influence of different pricing strategies with regard to their prescribing practices was not statistically significant.

## Discussion

Pharmaceutical companies spent more money on marketing than they did on research and development of new pharmaceuticals in the last decade [16], raising suspicions of potential conflicts of interest. Physicians have an important role in the pharmaceutical industry, speaking on behalf of the company and making judgments through prescribing drugs and influencing other physicians’ prescription practices, which is a significant aspect for pharmaceutical companies [17]. Because of personal interests and a conflict between money and ethics, the connection between physicians and pharmaceutical firms has always been contentious. According to several studies, the better medical representative )MR)s’ relationships with physicians are, the more likely doctors will be to write prescriptions for a specific drug [17]. In recent years, physician-pharmaceutical industry interactions and potential conflicts of interest have been discussed [18, 19]. Researchers and the general public are concerned that the practitioner’s core interest and the patient’s well-being, may be impacted by secondary interests such as perks provided by the pharmaceutical industry to physicians [20].

Wazana A. found that interactions between doctors and pharmaceutical representatives negatively impact doctor conduct and knowledge [21]. They claim to have a proclivity for irrational prescribing, a friendly attitude toward company representatives, a predilection for newer, more expensive treatments, and an inability to spot false medication claims. Furthermore, this effect on physicians’ practices and knowledge is dose-dependent: frequent interaction and the acceptance of presents, regardless of the value of the gift, [22] create a need to reciprocate, which influences medical judgment [23].

The physicians in our study demonstrated moderate to high influence by marketing mix strategies, accounting for 76.2% of the maximum attainable score for this sub-scale, with a median score of 141.0 (IQR: 118.0–156.0) out of 185. The ultimate goal of a pharmaceutical marketer is to create a product that stands out among competitors in the eyes of doctors. To attain a large market share, pharmaceutical businesses use four essential ingredients: promotion, place, product, and pricing [24]. Companies competing for better earnings by employing various strategies will result in increased medicine sales and market share in the municipality. Pharmaceutical businesses may utilise different marketing mix elements or apply them in different ways, which may have an impact on physicians’ prescribing patterns.

Physicians do not appear to be aware of how marketing efforts affect their practices. Korenstein found that clinicians are aware that industry advertising influences their colleagues’ prescribing but not their own [25]. A prior study in Jordan found that physicians received pharmaceutical company gifts with open arms and that pharmaceutical company gifts had a statistically significant impact on doctors’ prescription behaviour [7]. According to a previous study conducted in Yemen, the majority of physicians had positive interactions with medical personnel. Furthermore, the majority of physicians said they were under marketing pressure to prescribe specific medications [14]. Our research found that promotional techniques employed by pharmaceutical companies had a lower impact on physicians than other marketing mix factors like product strategy, distribution strategy, and pricing strategy. This was in contrast to a recent study conducted in Lebanon, which found that physicians who attended CME conferences, accepted MR visits, and received promotional medication pamphlets had their prescription patterns impacted [26]. According to a study conducted in Saudi Arabia, the frequency of MR visits was one of the most critical criteria influencing their prescribing judgments [27]. In addition, a prior study conducted in a south-eastern city in the United States of America found that drug brochures influenced physicians’ responses to marketing methods and that MR visits were high [28]. Another study in Lebanon in 2019 indicated that the majority of the physicians investigated modifying their prescribing behaviour, and it can easily be inferred that advertising methods have a detrimental impact on Lebanese physicians’ prescribing patterns [6]. It is worth noting, however, that different questionnaire tools were used in each of these studies, making direct comparison difficult.

The disparities among studies could be attributable to medical practitioners’ having difficulty keeping their knowledge and credentials up-to-date after graduating from medical school due to limited information sources. Lack of medication information was one of the causes that led to physicians prescribing medicines arbitrarily, according to a prior study done at Hawassa University teaching and referral hospitals in southern Ethiopia [29]. Prescribers rely on the information they find in their environment, for example, the information provided by MRs, because their sources of information are limited. According to an assessment, multinational firms’ information is frequently prejudiced and occasionally dangerously false [30]. This improper usage has major health and economic ramifications for the success of the national healthcare system, and incorporating this information into clinical areas is too complex [31]. The influence of diverse marketing mix elements on healthcare professionals is affected by differences in the surrounding environment from one country to the next.

In our study, physicians were more likely to be affected by pricing strategies implemented by pharmaceutical companies. This might be due to the economic situation in the country. The low income of many patients during the COVID-19 pandemic has had negative consequences. Previous systematic review studies identified factors affecting physicians’ prescribing practices among 33 studies all over the world and reported that treatment cost is one of the key variables affecting physicians’ prescribing practices [32].

Participating in company-sponsored CME, product launch meetings, and sponsorship for conference travel were the most widely agreed-upon promotional methods in terms of influencing physicians’ prescribing behaviours. This could be explained by the fact that pharmaceutical corporations carefully choose physicians and take them on tours of manufacturing facilities around the world. According to a prior study done in Pakistan, attending pharmaceutical company-sponsored vacations to touristic sites and visiting production plants enhanced physicians’ prescribing rates after they attended a company-sponsored event where all of their expenses were covered [33].

Quality of medicine, supportive proof of the efficacy of the medicine provided by the pharmaceutical business, and the release of novel innovations or combinations of drugs were the most frequently agreed-upon product strategies in terms of influencing physicians’ prescription practices. The country of origin of the pharmaceutical product, the method of administration, and the quality of the medicine are all crucial aspects that impact physicians’ prescribing patterns. These findings differed by country, with smaller effects as compared to a study done in Nairobi, which found that physicians were impacted in their prescribing behaviour by the style of medicine distribution in 85.8% of cases [34]. However, influences were larger when compared to a study conducted in Saudi Arabia, in which 46.2% of physicians were persuaded by a source from the drug’s manufacturer [35]. According to a study in Addis Ababa, the prescribing behaviour of 34.5% of physicians was impacted by the quality of medicine [36]. The disparity between studies could be explained by the fact that corporations invest a lot of money in innovation every year, and the new products are better than the old ones in terms of dosage, indication, side effects, and cost. After MRs promote the new generation of better medicine products, physicians choose them. It is difficult to diagnose because the town’s health institution lacks a fully equipped laboratory, so they prescribe medications with better coverage [37].

Inclusion of medicine on the hospital medicine list, pharmaceutical product availability, and availability of real-time product information from distribution intermediaries were the most commonly agreed-upon distribution strategies in terms of influencing physicians’ prescribing practices. In comparison to a study conducted in Nairobi, we found that medicine availability influenced physicians’ prescribing behaviour by 65.1%, the availability of the drug in hospital formularies was 45.4%, and the local agent representing the major firm was 31.8% [38]. This could be due to a variety of factors, such as people moving from one location to another for various reasons, disease spread increasing in the town, and diseases emerging that we have never seen before. Furthermore, the temperature difference between the towns and the city boosts disease and patient dissemination.

The price of the drug and the effectiveness of therapy, the price for full course therapy, and the price of medication in connection to quality were the most widely agreed-upon pricing techniques in terms of influencing physicians’ prescription practices. Medications’ cost is one of the most important elements that influences patients’ adherence to their therapy, and higher medication costs are linked to cost-related non-adherence [39]. According to a previous study, physicians believed that disclosure of the actual price of the product had a 68.4% influence on their prescribing behaviour, that the price of the drug and the effectiveness of therapy had an 82.3% influence, and that price competition among pharmaceutical companies had a 44.1% influence. This result was higher than that of a study conducted in Addis Ababa, which found that pharmaceutical pricing has a 23% impact on physicians’ prescribing practices [36]. In comparison, a study in Nairobi found that 56.4% of physicians’ prescription behaviour was influenced by drug prices in proportion to the severity of the indication, whereas 81.6% of physicians were influenced by drug prices concerning competing products [38]. According to the World Bank, Ethiopia is a low-income country, which could explain the disparity in findings [40]. Although the communities’ purchasing power differs, they prescribe medications based on the wealth of the patients. Physicians and patients are the pillars of decision-making in the current medical system when it comes to determining when treatment for a patient’s disease will begin. To determine the possibilities for selecting and prescribing pharmaceutical products for patients, physicians compare comparable costs, effects of various types of pharmaceuticals, and estimate the strengths and weaknesses of alternatives [41].

Working in private sector settings was an important predictor that increased the probability of physicians’ prescribing behaviour being influenced by marketing mix elements. This might be due to the healthcare system in the private sector being open; the pharmaceutical company MRs can easily meet individual doctors. Physicians who received their specialised education locally (in Jordan) were less likely to be affected by promotional tools provided by pharmaceutical companies (OR: 0.40; 95%CI: 0.21–0.77) (p < 0.01). This might be explained by the physicians’ already being educated about the medication by multiple visits from the MRs, who do not give the physicians time to forget about the medication. Physicians who have more than ten years of experience and those who work in the private sector were more likely to be affected by product strategy compared to others with (OR: 1.84; (95%CI: 1.13–3.01)) and (OR: 2.12; (95%CI: 1.34–3.36)) (p < 0.05), respectively. On the other hand, resident physicians were less likely to be influenced (OR: 0.39; (95%CI: 0.16–0.93)), (p < 0.05). This is expected, as more experienced physicians have more practical experience with the product and have tried its alternatives in different cases over the years. This provides a solid basis for them while prescribing medications to their new patients based on their confidence in the quality of their prescribed medications. Experienced physicians care first about their patients, even more than any pressure from pharmaceutical companies, so they give higher priority to the pharmaceutical entity itself as it is the different factor that improves the patient’s health outcome. Regarding physicians who work in the private sector compared to those who work in the governmental sector, the latter are allowed to prescribe specific brand names (the ones purchased through the tender), so if the brand name of a specific pharmaceutical company is not available in a governmental hospital, physicians will not be visited by MRs of that company since it has no commercial benefit. To some extent, this places those physicians under less promotional pressure from pharmaceutical companies, so they prescribe more independently.

Physicians who work in the private sector are more likely to be influenced by pharmaceutical companies’ distribution strategies (OR: 1.90; 95%CI: 1.20–3.01) (p < 0.01). However, internists were less likely to be influenced (OR: 0.44; 95%CI: 0.23–0.84) (p < 0.05). This is mainly because physicians in the governmental sector are allowed to prescribe specific brand names (the ones purchased through the tender), so if the brand name of a specific pharmaceutical company is not available in a governmental hospital, it will not be available inside it and patients will need to go to other pharmacies outside governmental hospitals and pay for it themselves.

We advise policymakers to organize a balanced relationship between pharmaceutical companies and physicians. Educational intervention and awareness programs for physicians should clarify each of the marketing mix strategies and to educate them on how to balance them. We advise those programs to all physicians, especially the high-risk groups that are affected by marketing mix strategies, like physicians in the private sector, physicians with ten years of experience or more, and those who have specialised outside Jordan. The government is advised to decrease the taxes on medications to decrease the overall cost for patients.

This study has several strengths. This is the first study to explore the influence of the four pharmaceutical marketing mix strategies (product, price, place, and promotion) on physicians’ prescribing practices in the Middle East region. We did not restrict our study population to specific specialities or settings (private or governmental), which will increase the generalisability of our findings. This study has some limitations. First, it was a cross-sectional study design that involved the distribution of online or paper-based surveys to the physicians. A self-administered questionnaire through an online platform could be prone to social desirability bias. However, owing to the current epidemic, everyone is using virtual meetings, social networking, and online platforms. As a result, we assume that we still targeted a well-representative sample. We did not reach our targeted sample size, as the total number of participants was 315, which represents 83.1% of our targeted sample size. Our pilot study involved 10 physicians only, which is lower than the preferred literature recommendation of being around 10% of the sample size. Around 61.0% of the study participants were aged younger than 35 years. Besides, 63.2% of the participants were males. The use of convenience sampling might have affected the generalisability of our findings as a nonprobability sampling technique was employed in this study. Therefore, our findings should be interpreted carefully.

## Conclusion

Physicians in our study were highly affected by marketing mix strategies, specifically price strategy. Policymakers should guarantee a balanced relationship with pharmaceutical companies and physicians. We should make sure that promotion strategies have a positive impact on patients’ health. Future studies exploring the effects of marketing mix elements on physicians’ prescribing practices for chronic disease medications and over-the-counter medications (OTC) are needed to check for differences. Interventional approaches to promote efficient interaction between pharmaceutical companies and physicians are warranted.

## Data Availability

The datasets used and/or analysed during the current study are available from the corresponding author on reasonable request.

## Abbreviations

**MR**: Medical Representative.
**SPSS**: Statistical Package for Social Sciences.
**SD**: Standard Deviation.
**MCQ**: Multiple Choices Question.
**MR**: Medical Representative.
